# Growing up or growing out? How soil pH and light affect seedling growth of a relictual rainforest tree

**DOI:** 10.1093/aobpla/plu011

**Published:** 2014-03-31

**Authors:** Catherine A. Offord, Patricia F. Meagher, Heidi C. Zimmer

**Affiliations:** 1 The Royal Botanic Gardens and Domain Trust, The Australian Botanic Garden, Mount Annan, NSW 2567, Australia; 2 Department of Forest and Ecosystem Science, University of Melbourne, Richmond, VIC 3121, Australia

**Keywords:** Araucariaceae, conifer, conservation, light, rainforest, relictual species, soil pH, threatened species, Wollemi pine.

## Abstract

Seedling growth rates can have important long-term effects on forest dynamics. Environmental variables such as light availability and edaphic factors can exert a strong influence on seedling growth. In the wild, seedlings of Wollemi pine (*Wollemia nobilis*) grow on very acid soils (pH ∼4.3) in deeply shaded sites (∼3 % full sunlight). To examine the relative influences of these two factors on the growth of young *W. nobilis* seedlings, we conducted a glasshouse experiment growing seedlings at two soil pH levels (4.5 and 6.5) under three light levels: low (5 % full sun), medium (15 %) and high (50 %). Stem length and stem diameter were measured, stem number and branch number were counted, and chlorophyll and carotenoid content were analysed. In general, increased plant growth was associated with increased light, and with low pH irrespective of light treatment, and pigment content was higher at low pH. Maximum stem growth occurred in plants grown in the low pH/high light treatment combination. However, stem number was highest in low pH/medium light. We hypothesize that these differences in stem development of *W. nobilis* among light treatments were due to this species' different recruitment strategies in response to light: greater stem growth at high light and greater investment in multiple stem production at low light. The low light levels in the *W. nobilis* habitat may be a key limitation on stem growth and hence *W. nobilis* recruitment from seedling to adult. Light and soil pH are two key factors in the growth of this threatened relictual rainforest species.

## Abstract (precis)

Our aim was to uncover the drivers of seedling growth in a rare rainforest conifer. *Wollemia nobilis* is limited to canyons, characterized by deeply shaded understories and acid soils. In a glasshouse experiment, we grew seedlings at a range of pH and light levels. Growth increased with increasing light, and was higher at low pH, regardless of light. Number of stems, however, was greatest in lower light. *Wollemia nobilis* seedlings may vary their architecture - growing up when light is high, and growing out when light is lower. Nevertheless, low light is likely the key limitation of *W. nobilis* growth in the wild.

## Introduction

Light is a fundamental factor limiting the growth and survival of seedlings in closed forests (Chazdon *et al.* 1996; Whitmore 1996; Nicotra *et al.* 1999). Recent work has suggested that soil pH may be of equal or greater importance than light (Holste *et al.* 2011). Species-specific growth and survival responses to resources, particularly soil and light, play a key role in determining forest composition (Grubb 1977; Davies *et al.* 2005; Holste *et al.* 2011). Central to understanding tree species persistence is an understanding of their strategy for recruitment from understorey to canopy. Tree species range from those that are slow growing and shade tolerant, to fast-growing pioneers that typically dominate the post-disturbance environment (Denslow 1980; Whitmore 1989). It is competition for resources, governed by differences in survival and growth rate, that results in forest changes through time (i.e. stand development).

The influence of soil in defining species and plant community distributions is well known (Beadle 1954; Russo *et al.* 2008). Soil pH governs many plant–soil chemical relations, particularly the availability of micronutrients and toxic ions, due to its influence on solubility. At low pH, the availability of essential micronutrients Fe, Mn, Cu and Zn is increased, as is the availability of potentially toxic Al and Mn (Atwell *et al.* 2003). Alternatively, the availability of P and Mo decreases. High-pH soils, however, are high in Cr, Co, Ni, Fe and Mg, and deficient in N, P, K and Ca (Atwell *et al.* 2003). Plants with optimal growth and survival below and above pH 5–7 are known as acidophiles and calciphiles, respectively (Ehrenfeld *et al.* 2005); these plants employ strategies to avoid or tolerate otherwise suboptimal conditions. For example, acidophiles avoid the stresses of nutrient deficiency by conservation of minerals via slow growth, high storage in seeds, high root surface area and relationships with rhizosphere microorganisms (Marschner 1991). Soil pH can also influence plant community dynamics: low pH can prevent invasion of exotic species into native acid-tolerant plant communities (Thompson *et al.* 2001), while some species are apparently restricted to high-pH environments (e.g. ultramafics in New Caledonia; Jaffré 1992).

Wollemi pine (*Wollemia nobilis*, Araucariaceae) is a rare conifer with a highly restricted distribution. Araucariaceae is a family with origins in the early Triassic (Kershaw and Wagstaff 2001), and the earliest fossil record of *Wollemia* is from 91 million years ago (*Dilwynites* pollen; Macphail *et al.* 1995). *Wollemia nobilis* has unique architecture with only first-order plagiotropic branches and is capable of producing multiple stems without injury (Hill 1997). The plagiotropic branches can grow up to 150 cm long and are shed whole, forming a dense litter layer. Branches are short-lived (<15 years) and have either adult or juvenile leaf types above and below the rainforest canopy, respectively. Wollemi pine grows in several small stands in the Wollemi National Park, part of the Greater Blue Mountains World Heritage area, New South Wales, Australia (Jones *et al.* 1995; NSW Department of Environment and Conservation 2006). Wollemi National Park has predominantly sandstone geology (Jones *et al.* 1995), which is typically associated with acid soils (Binkley and Fisher 2000). *Wollemia nobilis* exists at the base, and on low terraces, of deep narrow canyons within a warm temperate rainforest community with co-dominant *Ceratopetalum apetalum* (Benson and Allen 2007). Recruitment from seed to adult is rare, even though seed production is relatively high (NSW Department of Environment and Conservation 2006). Fewer than 100 adult trees have been discovered, and some 300 seedlings have been observed, the majority under 500-mm stem length. Height growth of *W. nobilis* seedlings is very slow in the wild (5–20 mm per year; Zimmer *et al.* 2014). The presence of seedlings indicates that *W. nobilis* is capable of producing viable seed (Offord *et al.* 1999). Yet lack of intermediate-sized trees (i.e. 5–20 m) and slow growth indicates that there are other factors limiting their establishment (Whitmore and Page 1980).

The overarching aim of this study was to explore the relative importance of light and soil pH in determining *W. nobilis* seedling success. To do this we investigated *W. nobilis* growth in response to the natural light and soil pH conditions where *W. nobilis* grows in the wild, and then to a wider range of light and pH conditions, in a glasshouse experiment.

## Methods

### Field observations

A preliminary study of the soil characteristics associated with the areas in which *W. nobilis* grows indicated that the pH is very acidic (∼pH 4) (NSW Department of Environment and Conservation 2006). Soil samples (10 × 500 g) were collected adjacent to *W. nobilis* seedlings growing in the wild. Soil pH was measured in water 1 : 2.

Photosynthetic photon flux density (PPFD) was measured around 42 *W. nobilis* seedlings with a hand-held Licor quantum light meter around midday on two typical sunny days in summer. These were compared with full-sun light meter readings in nearby areas.

### Glasshouse treatments

*Wollemia nobilis* seeds collected from multiple trees in the wild were germinated in Petri dishes in growth cabinets set at 24 °C (Offord and Meagher 2001). Freshly germinated seedlings were grown in 75-mm (0.44-L) pots containing steam-pasteurized peat and sand (1 : 2 v/v) at pH 4.5 or adjusted to pH 6.5 with lime and dolomite (1 : 1 w/w). When seedlings were 4–5 months of age they were potted in 140-mm (1.5-L) pots containing the same potting mix and pH treatments with the addition of the fertilizer Nutricote Total N_13_ (13 : 5.7 : 10.8 N : P : K) 270 day type added to the mix at a rate of 3 g L^−1^ prior to steam pasteurization (time zero). Pasteurization was undertaken such that it did not affect fertilizer release rates (temperatures did not exceed 60 °C for >30 min). Plants were fertilized and re-potted after the 12-month assessment. Plants were watered daily or as needed.

In a glasshouse at the Australian Botanic Garden, Mount Annan (ABGMA, 34°05′S, 150°47′E), plants were randomly assigned to areas with 5, 15 or 50 % full sunlight (low, medium or high relative light). This was achieved by using different grades of shade cloth with the same wavelength transmission properties, in addition to light attenuated by the glasshouse. The light at plant level relative to ambient was determined using a pyranometer (Environdata P/L). The temperatures within the glasshouse were controlled to a mean of 24 °C (standard error [SE] = 3 °C) during the day and 16 °C (SE = 3 °C) at night. There were 20 replicate potted plants of each light and pH treatment combination (in a full factorial design). Plants were randomly positioned, and randomly re-positioned after each measurement, within each light treatment.

**Figure 1.**
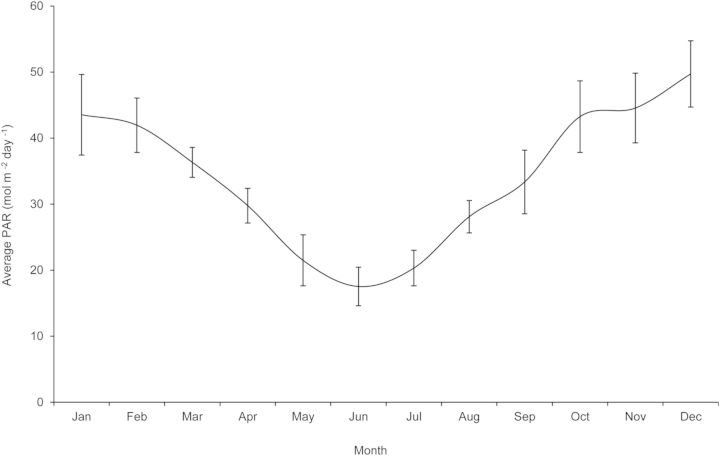
Average daily PAR (mol m^−2^) received at the Australian Botanic Garden Mount Annan (5-year average 1998–2002) (error bars = SE).

Plant growth characteristics were recorded at 6, 12 and 24 months after time zero. This included number and length of orthotropic (vertical) stems and plagiotropic (horizontal) branches, diameter at the base of the plant and general health characteristics. Destructive methods could not be used on the seedlings because of their rarity. Stem length was calculated as total length of all stems. Measurements were taken over 24 months because in a similar study of *Araucaria angustifolia*, measurements were made after only 4 months of growth, at which time there were no differences in the stem length or chlorophyll variables measured (Duarte and Dillenburg 2000). Average daily accumulation of incident photosynthetically active radiation (PAR) by month was calculated using 5 years of solar radiation data for ABGMA collected using a pyranometer (Fig. 1). Solar radiation (energy) was converted to PAR (quanta) using the correction factor *c* = 2.3 (Monteith and Unsworth 1990).

### Pigment extraction and concentration

At 24 months four leaf samples taken from the new leaves of three plants per treatment combination were analysed for chlorophyll (chlorophyll *a* and chlorophyll *b*, protochlorophyll) and carotenoids (Chen *et al.* 1998).

### Statistical analysis

Two-way analysis of variance (ANOVA) was conducted on all variables (SYSTAT; SPSS Inc.). Where there were no significant interactions, *post hoc* tests, specifically least significant difference (LSD), were undertaken. Where significant interactions were found, one-way ANOVAs and/or *t*-tests were conducted on the variables, and these results, and associated LSDs, were reported. Data for stem and branch number were log-transformed to normalize the data for analysis. Untransformed data are presented in the tables.

## Results

### Field observations

Mean soil pH in the field was 4.32 (SE = 0.12, *n* = 10) in water. Light levels at *W. nobilis* seedlings in the field were highly variable and were as low as 1 % of full sunlight even at the brightest time of the day. Light penetration into the canyon was restricted by its depth, the angle of the sun and the dense canopy of other tree species growing within it. On the canyon floor, PPFD at approximately midday on a sunny day in February averaged 60 μmol m^−2^ s^−1^ (SE = 9, *n* = 43), which represents around 3 % of full sunlight measured in adjacent open areas (mean = 2000 μmol m^−2^ s^−1^, SE = 163, *n* = 4).

### Growth measurements

**Figure 2.**
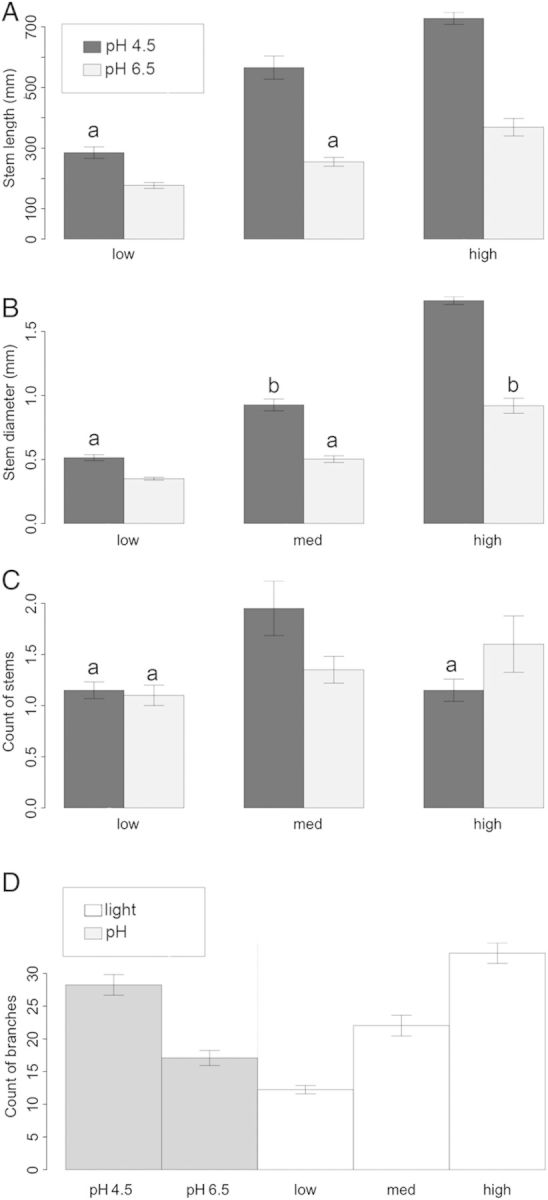
Growth characteristics of *W. nobilis* seedlings grown at low, medium and high light in potting mix at pH 4.5 or 6.5 for 24 months*.* (A) Stem length, (B) stem diameter and (C) stem count means taken across combined light and pH treatments (±SE). (D) Mean branch count is presented separately for (i) light treatments and (ii) pH treatments (±SE). Within each response variable, means sharing the same letter are not significantly different by LSD_5%_. Note: *y*-axis varies.

For clarity, only the 24-month data are presented for growth and pigment variables. Significant interactions between pH and light were found for stem length, stem diameter and number of stems (*P* < 0.05; Table 1); therefore, simple main effects were investigated for these variables (Table 1, Fig. 2). At the higher pH, the light level made little difference to the stem length of the plant compared with the large difference made by increased light in low-pH treatments, particularly at medium light. The significant interaction of light and pH for stem number can be accounted for by the higher number of stems found in the medium-light/low-pH treatment. Branch number was significantly higher at low pH. The increases in branch number with increasing light were approximately proportional to the increases in stem length.

**Table 1.** Analysis of variance of measured *W. nobilis* seedling characters (growth and leaf pigment) according to treatment variables (light and pH). Where there were significant interactions between the treatments, simple main effects are presented (lower table). **P < 0.01; *P < 0.05; NS, not significant; ^#^*P* = 0.050.

*F*-test significance	Stem length (mm)	Diameter at base (mm)	Number of stems	Number of branches	Chlorophyll *a*	Chlorophyll *b*	Chlorophyll *a* + *b*	Chlorophyll *a*/*b*	Carotenoids/chlorophyll	Protochlorophyll
Light	**	**	*	**	**	NS	*	**	NS	NS
pH	**	**	NS	**	**	**	**	**	*	**
Light × pH	*	**	*	NS	NS	NS	NS	NS	NS	NS
Low light	**	**	NS							
Med light	**	**	NS^#^							
High light	**	**	NS							
pH 4.5	**	**	**							
pH 6.5	**	**	NS

### Leaf pigment content

No significant interactions between light and pH were detected for the pigment concentrations (*P* > 0.05; Table 1). The concentrations of chlorophyll *a*, chlorophyll *a* + *b* and chlorophyll *a*/*b* were significantly associated with light (*P* < 0.05; Table 1). Chlorophyll *a* and chlorophyll *b* were higher in low light. The highest chlorophyll *a* + *b* concentration was in plants under the higher light treatments. The chlorophyll *a*/*b* ratio was also significantly higher at low and medium light.

**Figure 3.**
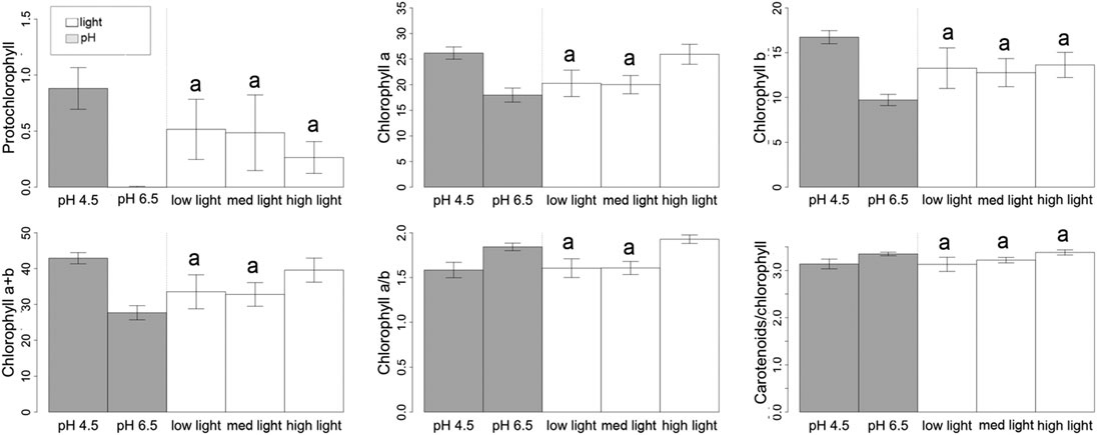
Pigment concentration (μg cm^−2^) or ratio in leaves of *W. nobilis* seedlings grown at low, medium and high light in potting mix at pH 4.5 or 6.5 for 24 months (mean ± SE). (A–F) Means for pH and light treatments (means sharing the same letter are not significantly different by LSD_5%_). Note: *y*-axis varies.

The concentrations of chlorophyll *a* and chlorophyll *a* + *b* in the leaves of *W. nobilis* were also highly significantly associated with pH (*P* < 0.01): there were higher concentrations at the lower pH (Table 1, Fig. 3). The chlorophyll *a*/*b* ratio was significantly higher in the high-pH treatments, compared with low-pH treatments. The protochlorophyll levels were significantly higher in the low-pH treatments, compared with high-pH treatments. In line with this, chlorophyll *a* and chlorophyll *b* were significantly higher at low pH. Protochlorophyll tended to be higher at low- and medium-light treatments, but this variation was not significant. The carotenoid-to-chlorophyll ratio was higher in the high-pH and low-light treatments, which was reflected in the generally chlorotic appearance of these plants.

## Discussion

*Wollemia nobilis* is an acidophile. Growth of seedlings was maximal at a soil pH considered suboptimal for many species (pH 4.5; Handreck and Black 2002). *Wollemia nobilis* growth also increased with increased light, but this response was moderated by pH—higher pH resulted in growth suppression. Moreover, the chlorophyll content (chlorophyll *a* + *b*) of *W. nobilis* leaves was higher in the low soil pH treatments, indicating increased growth and, perhaps, potential for growth.

Acid soils are a feature of natural stands of Araucariaceae, particularly *Agathis australis* (Mirams 1957; Bieleski 1959; Ecroyd 1982; Weaver 1988; Wyse 2012), and Araucariaceae plantations (Curlevski *et al.* 2010). Changes in soil acidity under different plants can be due to differences among species in nutrient accumulation (Alban 1982; Finzi *et al.* 1998), nitrogen fixation (van Miegroet and Cole 1984), litter chemical composition (Ovington 1953; Alban 1982; Finzi *et al.* 1998) and the stimulation of mineral weathering (Tice *et al.* 1996). The source of acid soils associated with Araucariaceae is hypothesized to be litterfall (Bieleski 1959; Silvester and Orchard 1999). Acid soils (pH < 7) are also common in eastern New South Wales, but soils of pH ≤4.5 are much less common (Helyar *et al.* 1990). Sandstone-derived soils are also typically acidic (Binkley and Fisher 2000). Because we did not test the soils beyond the *W. nobilis* stand, we cannot say what effect (if any) *W. nobilis* has on the soil. Instead, our results suggest that low pH, and associated changes in soil nutrients, is likely to be a major factor in enhancing the growth of established *W. nobilis* seedlings. Indeed, low pH may also indirectly benefit *W. nobilis* by reducing competition from other species not adapted to acid soils.

*Wollemia nobilis* seedling growth increased with increasing light availability. Seedling growth was suppressed by low light (5 %), resulting in stem lengths half that attained by the high-light (50 %) treatment at 24 months. Previous studies have demonstrated that rainforest species, including *C. apetalum*, have lower growth at high light, but higher mean biomass accumulation at low light, when compared with eucalypt or ecotone species (Barrett and Ash 1992). This study revealed that while stem length increased with increased light, the number of stems was highest in medium light. We hypothesize that this species has a low-light strategy of producing more stems and a high-light (i.e. large gap) strategy to invest more in growth of primary stems. However, it appears that multiple stem production in low light increases substantially above a threshold light level, between 5 and 15 %; this threshold is yet to be defined.

*Wollemia nobilis* seedlings respond to increased light by increasing growth, but they can also grow slowly and survive in low light. In low light, *W. nobilis* can maintain its photosynthetic apparatus in a state in which it can take advantage of changes in light levels, indicated by the chlorophyll pigment concentrations and ratio. However very low light (and associated low growth) in the wild may contribute to seedling mortality by fungal pathogens, herbivory, litterfall and drought, in line with models of increased juvenile risk in slow-growing species (Bond 1989).

Young *W. nobilis* leaves are adapted to shaded understorey conditions, maximizing light interception by arranging leaves in a single plane, while adult foliage is arranged in two ranks to capture light from all around (Hill and Brodribb 2003). Moreover, *W. nobilis* juveniles had a high proportion of branches to stems; this is typical of many understorey species, and demonstrates structural flexibility (Givnish 1988). This study indicates that *W. nobilis* has the ability to produce multiple leaders at all light levels, but particularly at medium-light and low-pH soil growth conditions. The difference among light treatments, in the production of multiple stems, was only apparent at the 24-month measurement (compared with the 12-month measurement; data not presented). This is comparable to the growth lag phase, which has been observed previously in *W. nobilis* (Offord *et al.* 1999). Hence, *W. nobilis* can take advantage of light gaps by extending leaders, in effect seeking light and then producing the branches at a later time to maximize growth in the gap (Givnish 1988).

The maximum light treatment used in our study was 50 %, rather than 100 %, full sun. Previous research has shown that young *Agathis* trees can be damaged by full sunlight (Whitmore 1977), and along with other temperate rainforest species, *Agathis* have been shown to have lower chlorophyll concentrations in full sunlight compared with trees grown in medium and heavy shade (Langenheim *et al.* 1984; Read 1985). Moreover, photoinhibition may occur before obvious leaf damage. *Wollemia nobilis* is highly susceptible to heat stress (compared with other Araucariaceae species; Offord 2011), and anecdotal observations of young *W. nobilis* (<5 years) suggest that the leaves can become chlorotic under full sun. The quality of light in the canopy, especially the ratio of red to far red, may also influence the growth of shade-adapted *Agathis* species (Warrington *et al.* 1988). Although this study has found a positive correlation between light and growth in *W. nobilis* seedlings, this effect is likely to be curtailed at higher light availabilities due to light saturation.

The positive effects of light on growth were moderated by pH. Growth of stems (height and diameter), and stem number, was highest in the low-pH treatments, regardless of light. In contrast, in the high-pH treatments, increased light was associated with increased growth. While both pH and light affected pigments, there was no modulating effect detected. Over the range of light availabilities assessed, the chlorophyll content and the *a* to *b* ratio varied little, compared with the variation associated with pH. Protochlorophyll is a precursor to chlorophyll and is readily converted with light exposure (Lancer *et al.* 1976; Huq *et al.* 2004). Protochlorophyll was significantly higher in the low-pH treatment. The direct effects of pH on protochlorophyll are unknown, but low levels (in the high-pH treatment) suggest that the plants would be less able to take advantage of increases in light. Higher carotenoid-to-chlorophyll ratios in the high-pH treatment may also reflect growth suppression. Soil fertility (influenced by pH) can also affect plants' capacity to capture light (Baltzer and Thomas 2005). However, the combined effects of light and pH were not significant in defining pigment concentrations in this study.

### Implications for *W. nobilis* stand dynamics

The need for open canopy conditions, with >10 % light (i.e. large canopy gaps), for recruitment of juveniles to canopy trees is evident in a number of Araucariaceae species (Bergin and Kimberley 1987; Enright *et al.* 1993; Fincke and Paulsch 1995; Rigg *et al.* 1998). Furthermore, infrequent large-scale landscape disturbance is required for substantial recruitment of other Araucariaceae species (Ogden *et al.* 1992; Burns 1993; Enright *et al.* 1999). Likewise, very low rates of seedling recruitment are a feature of mature undisturbed Araucariaceae stands (in association with increasing dominance of angiosperm tree species; Enright *et al.* 1999). The demonstrated increase in *W. nobilis* growth with light availability is consistent with growth responses observed in other Araucariaceae species.

Our results imply that canopy gaps may be required for significant increases in stem length and hence recruitment from *W. nobilis* juveniles to canopy trees. How much light required is unknown, but positive responses to 50 % light availability (i.e. large gaps) were recorded in this study. If *W. nobilis* growth and recruitment to larger size classes is dependent on light, this may explain the lack of intermediate-sized *W. nobilis* juveniles in the wild, where *W. nobilis* is competing with rainforest angiosperms (*sensu* Enright *et al.* 1999). *Wollemia nobilis* response to increased light availability after long-term suppression in the shade is also unknown. The self-coppicing habit of *W. nobilis* allows it to survive in low light (Givnish 1988) and may aid recovery from disturbance (Dietze and Clark 2008). Its architecture may also mean that it can respond quickly (change morphologically) to intercept light (Canham 1989; Whitmore 1989), similar to *A. angustifolia*, which can quickly colonize gaps from both seedling banks and resprouts of damaged trees via plastic growth patterns (Duarte *et al.* 2002).

The ability of *W. nobilis* to respond to increased light was strongly moderated by soil pH. Although there is evidence to suggest that Araucariaceae can modify soil pH, we suggest that pH is unlikely to be limiting *W. nobilis* success in the wild as acid soils are widespread in eastern New South Wales; hence the question remains, what factors limit the distribution of *W. nobilis* in the wild? Despite the strong limiting effect of pH on growth demonstrated in this study, light availability (i.e. the presence of large canopy gaps) may be more limiting in the wild.

### Implications for conservation of *W. nobilis*

Understanding the climatic and edaphic factors governing plant growth is important for the conservation management of species in the wild, and especially species like *W. nobilis* that have restricted distributions and are under threats such as disease, fire and climate change. Where management of wild stands cannot provide sufficient protection, translocation of threatened species is one complementary conservation measure (Vallee *et al.* 2004). Selection of suitable soil types and light regimes provided by topography, aspect and vegetation assemblages is essential to translocation success. Additionally, optimal growth of plants *ex situ*, in gardens or germplasm collections, necessitates knowledge of fundamental growth requirements. This and several other studies partially provide this knowledge for *W. nobilis* (vide Offord 2011). Importantly, our results indicate that *W. nobilis* recruitment may be light limited in the wild. Our results also suggest that *W. nobilis* translocation efforts should be focused on sites with low-pH soils and outside the deeply shaded conditions of a closed rainforest.

## Conclusions

Seedling growth responses to varying light regimes suggest that *W. nobilis* is a shade-tolerant, gap-responding species: tolerating low light and increasing growth at higher light. However, this response is strongly moderated by soil pH; *W. nobilis* growth is significantly enhanced on low-pH soils. While these factors clearly influence seedling growth of this species, at least under glasshouse conditions, other factors, such as drought, herbivory and microbial interactions, may also strongly influence the recruitment of this species in the wild.

## Sources of Funding

Our work was funded by the Royal Botanic Gardens and Domain Trust, Sydney.

## Contributions by the Authors

C.O. and P.M. were responsible for the experimental design. P.M. conducted the experimental components including data collection. C.O. and H.Z. analysed data and wrote the manuscript.

## Conflicts of Interest Statement

None declared.

## References

[PLU011C1] Alban DH (1982). Effects of nutrient accumulation by aspen, spruce, and pine on soil properties. Soil Science Society of America Journal.

[PLU011C2] Atwell B, Kriedemann P, Turnbull C (2003). Plants in action: adaptations in nature, performance in cultivation.

[PLU011C3] Baltzer JL, Thomas SC (2005). Leaf optical responses to light and soil nutrient availability in temperate deciduous trees. American Journal of Botany.

[PLU011C4] Barrett D, Ash J (1992). Growth and carbon partitioning in rainforest and eucalypt forest species of south coastal New South Wales, Australia. Australian Journal of Botany.

[PLU011C5] Beadle N (1954). Soil phosphate and the delimitation of plant communities in eastern Australia. Ecology.

[PLU011C6] Benson J, Allen C (2007). Vegetation associated with *Wollemia nobilis* (Araucariaceae). Cunninghamia.

[PLU011C7] Bergin DO, Kimberley MO (1987). Establishing kauri in a pine stand and in scrub. New Zealand Journal of Forestry Science.

[PLU011C8] Bieleski RL (1959). Factors affecting growth and distribution of kauri (*Agathis australis* Salisb.). III. Effect of temperature and soil conditions. Australian Journal of Botany.

[PLU011C9] Binkley D, Fisher R (2000). Ecology and management of forest soils.

[PLU011C10] Bond W (1989). The tortoise and the hare: ecology of angiosperm dominance and gymnosperm persistence. Biological Journal of the Linnean Society.

[PLU011C11] Burns BR (1993). Fire-induced dynamics of *Araucaria araucana*—*Nothofagus antartica* forest in the southern Andes. Journal of Biogeography.

[PLU011C12] Canham CD (1989). Different responses to gaps among shade-tolerant tree species. Ecology.

[PLU011C13] Chazdon RL, Pearcy RW, Lee DW, Fetcher N, Mulkey SS, Chazdon RL, Smith AP (1996). Photosynthetic responses of tropical forest plants to contrasting light environments. Tropical forest plant ecophysiology.

[PLU011C14] Chen DM, Keiper FJ, De Filippis LF (1998). Physiological changes accompanying the induction of salt tolerance in *Eucalyptus microcorys* stems in tissue culture. Journal of Plant Physiology.

[PLU011C15] Curlevski NJA, Xu Z, Anderson IC, Cairney JWG (2010). Converting Australian tropical rainforest to native Araucariaceae plantations alters soil fungal communities. Soil Biology and Biochemistry.

[PLU011C16] Davies S, Tan S, LaFrankie JV, Potts MD (2005). Soil-related floristic variation in a hyperdiverse dipterocarp forest. Pollination ecology and the rain forest.

[PLU011C17] Denslow JS (1980). Patterns of plant species diversity during succession under different disturbance regimes. Oecologia.

[PLU011C18] Dietze MC, Clark JS (2008). Changing the gap dynamics paradigm: vegetative regeneration control on forest response to disturbance. Ecological Monographs.

[PLU011C19] Duarte LS, Dillenburg LR (2000). Ecophysiological responses of *Araucaria angustifolia* (Araucariaceae) seedlings to different irradiance levels. Australian Journal of Botany.

[PLU011C20] Duarte LS, Dillenburg LR, Rosa LMG (2002). Assessing the role of light availability in the regeneration of *Araucaria angustifolia* (Araucariaceae). Australian Journal of Botany.

[PLU011C21] Ecroyd CE (1982). Biological flora of New Zealand. 8. *Agathis australis*. D.Don. Lindl. (Araucariaceae) Kauri. New Zealand Journal of Botany.

[PLU011C22] Ehrenfeld JG, Ravit B, Elgersma K (2005). Feedback in the plant–soil system. Annual Review of Environment and Resources.

[PLU011C23] Enright NJ, Bartlett RM, De Freitas CR (1993). Patterns of species composition, recruitment, and growth within canopy gaps in two New Zealand kauri (*Agathis australis*) forests. New Zealand Journal of Botany.

[PLU011C24] Enright NJ, Ogden J, Rigg LS (1999). Dynamics of forests and Araucariaceae in the western Pacific. Journal of Vegetation Science.

[PLU011C25] Fincke M, Paulsch A (1995). The ecological strategy of *Araucaria araucana*. Flora.

[PLU011C26] Finzi AC, Canham CD, van Breemen N (1998). Canopy tree–soil interactions within temperate forests: species effects on pH and cations. Ecological Applications.

[PLU011C27] Givnish TJ (1988). Adaptation to sun and shade: a whole-plant perspective. Journal of Plant Physiology.

[PLU011C28] Grubb PJ (1977). The maintenance of species-richness in plant communities: the importance of the regeneration niche. Biological Reviews.

[PLU011C29] Handreck K, Black N (2002). Growing media for ornamental plants and turf.

[PLU011C30] Helyar K, Cregan P, Godyn D (1990). Soil acidity in New-South-Wales—current pH values and estimates of acidification rates. Soil Research.

[PLU011C31] Hill KD (1997). Architecture of the Wollemi pine (*Wollemia nobilis*, Araucariaceae), a unique combination of model and reiteration. Australian Journal of Botany.

[PLU011C32] Hill RS, Brodribb TJ (2003). Evolution of conifer foliage in the southern hemisphere. Acta Horticulturae.

[PLU011C33] Holste EK, Kobe RK, Vriesendorp CF (2011). Seedling growth responses to soil resources in the understory of a wet tropical forest. Ecology.

[PLU011C34] Huq E, Al Sady B, Hudson M, Kim C, Apel K, Quail PH (2004). Phytochrome-interacting factor 1 is a critical bHLH regulator of chlorophyll biosynthesis. Science.

[PLU011C35] Jaffré T (1992). Floristic and ecological diversity of the vegetation on ultramafic rocks in New Caledonia. *The Vegetation of Ultramafic (Serpentine) Soils: Proceedings of the First International Conference on Serpentine Ecology*.

[PLU011C36] Jones WG, Hill KD, Allen JM (1995). *Wollemia nobilis*, a new living Australian genus and species in the Araucariaceae. Telopea.

[PLU011C37] Kershaw P, Wagstaff B (2001). The southern conifer family Araucariaceae: history, status, and value for paleoenvironmental reconstruction. Annual Review of Ecology and Systematics.

[PLU011C38] Lancer HA, Cohen CE, Schiff JA (1976). Changing ratios of phototransformable protochlorophyll and photochlorophyllide of bean seedlings developing in the dark. Plant Physiology.

[PLU011C39] Langenheim JH, Osmond CB, Borrks A, Ferrar PJ (1984). Photosynthetic responses to light in seedlings of selected Amazonian and Australian rainforest tree species. Geologia.

[PLU011C40] Macphail M, Hill K, Partridge A, Truswell E, Foster C (1995). Wollemi Pine ‘old pollen records for a newly discovered genus of gymnosperm. Geology Today.

[PLU011C41] Marschner H (1991). Mechanisms of adaptation of plants to acid soils. Plant and Soil.

[PLU011C42] Mirams RV (1957). Aspects of the natural regeneration of the kauri (*Agathis australis* Salisb.). Transactions of the Royal Society of New Zealand.

[PLU011C43] Monteith JL, Unsworth M (1990). Principles of environmental physics.

[PLU011C44] Nicotra AB, Chazdon RL, Iriarte SVB (1999). Spatial heterogeneity of light and woody seedling regeneration in tropical wet forests. Ecology.

[PLU011C45] NSW Department of Environment and Conservation (2006). *Wollemia nobilis* (Wollemi pine) recovery plan.

[PLU011C46] Offord CA (2011). Pushed to the limit: consequences of climate change for the Araucariaceae: a relictual rain forest family. Annals of Botany.

[PLU011C47] Offord CA, Meagher PF (2001). The effects of temperature, light and stratification on seed germination of Wollemi pine (*Wollemia nobilis*, Araucariaceae). Australian Journal of Botany.

[PLU011C48] Offord CA, Porter CL, Meagher PF, Errington G (1999). Sexual reproduction and early plant growth of the Wollemi pine (*Wollemia nobilis*), a rare and threatened Australian conifer. Annals of Botany.

[PLU011C49] Ogden J, Wilson A, Hendy C, Newnham RM (1992). The late Quaternary history of kauri (*Agathis australis*) in New Zealand and its climatic significance. Journal of Biogeography.

[PLU011C50] Ovington JD (1953). Studies of the development of wood-land conditions under different trees. I. Soil pH. Journal of Ecology.

[PLU011C51] Read J (1985). Photosynthetic and growth responses to different light regimes of the major canopy species of Tasmanian cool temperate rainforest. Australian Journal of Ecology.

[PLU011C52] Rigg LS, Enright NJ, Jafffré T (1998). Stand structure of the emergent conifer *Araucaria laubenfelsii*, in maquis and rainforest, Mont Do, New Caledonia. Australian Journal of Ecology.

[PLU011C53] Russo SE, Brown P, Tan S, Davies S (2008). Interspecific demographic trade-offs and soil-related habitat associations of tree species along resource gradients. Journal of Ecology.

[PLU011C54] Silvester WB, Orchard TA (1999). The biology of kauri (*Agathis australis*) in New Zealand. Production, biomass, carbon storage, and litter fall in four forest remnants. New Zealand Journal of Botany.

[PLU011C55] Thompson K, Hodgson JG, Grime JP, Burke MJW (2001). Plant traits and temporal scale: evidence from a 5-year invasion experiment using native species. Journal of Ecology.

[PLU011C56] Tice KR, Graham RC, Wood HB (1996). Transformations of 2:1 phyllosilicates in 41-year-old soils under oak and pine. Geoderma.

[PLU011C57] Vallee L, Hogbin T, Monks L, Makinson B, Matthes M, Rossetto M (2004). Guidelines for the translocation of threatened plants in Australia.

[PLU011C58] Van Miegroet H, Cole DW (1984). The impact of nitrification on soil acidification and cation leaching in a red alder ecosystem. Journal of Environmental Quality.

[PLU011C59] Warrington IJ, Rook DA, Morgan DC, Turnbull HL (1988). The influence of simulated shadelight and daylight on growth, development and photosynthesis of *Pinus radiata*, *Agathis australis* and *Dacrydium cuperssinum*. Plant, Cell and Environment.

[PLU011C60] Weaver SA (1988). Soil differences between secondary and old growth *Agathis macrophylla* forest at Nadarivatu, Fiji. Tuatara.

[PLU011C61] Whitmore TC (1977). A first look at Agathis.

[PLU011C62] Whitmore TC (1989). Canopy gaps and the two major groups of forest trees. Ecology.

[PLU011C63] Whitmore TC, Swaine MD (1996). A review of some aspects of tropical rain forest seedling ecology with suggestions for further enquiry. The ecology of tropical forest tree seedlings.

[PLU011C64] Whitmore TC, Page CN (1980). Evolutionary implications of the distribution and ecology of the tropical conifer *Agathis*. New Phytologist.

[PLU011C65] Wyse SV (2012). Growth responses of five forest plant species to the soils formed beneath New Zealand kauri (*Agathis australis*). New Zealand Journal of Botany.

[PLU011C66] Zimmer HC, Auld TD, Benson J, Baker PJ (2014). Recruitment bottlenecks in the rare Australian conifer. *Wollemia nobilis*. Biodiversity and Conservation.

